# The correlation between the length of repetitive domain and mechanical properties of the recombinant flagelliform spidroin

**DOI:** 10.1242/bio.022665

**Published:** 2017-01-26

**Authors:** Xue Li, Chang-Hua Shi, Chuan-Long Tang, Yu-Ming Cai, Qing Meng

**Affiliations:** 1 College of Environmental Science and Engineering, Donghua University, Shanghai 201620, People's Republic of China; 2 Institute of Biological Sciences and Biotechnology, Donghua University, Shanghai 201620, People's Republic of China

**Keywords:** Flagelliform silk, Wet-spinning, Synthetic fiber, Mechanical properties, Fiber structure

## Abstract

Spider silk is an attractive biopolymer with numerous potential applications due to its remarkable characteristics. Among the six categories of spider silks, flagelliform (Flag) spider silk possesses longer and more repetitive core domains than others, therefore performing the highest extensibility. To investigate the correlation between the recombinant spidroin size and the synthetic fiber properties, four recombinant proteins with different sizes [N-Scn-C (*n*=1-4)] were constructed and expressed using IMPACT system. Subsequently, different recombinant spidroins were spun into fibers through wet-spinning via a custom-made continuous post-drawing device. Mechanical tests of the synthetic fibers with four parameters (maximum stress, maximum extension, Young's modulus and toughness) demonstrated that the extensibility of the fibers showed a positive correlation with spidroin size, consequently resulting in the extensibility of N-Sc4-C fiber ranked the highest (58.76%) among four fibers. Raman data revealed the relationship between secondary structure content and mechanical properties. The data here provide a deeper insight into the relationship between the function and structure of Flag silk for future design of artificial fibers.

## Abstract (precis)

**Summary:** A study of the relationship between the structure and property of synthetic spider silk-like fibers, which aims to aid with the designing of functional artificial fibers.

## INTRODUCTION

Orb-weaving spiders can produce up to six kinds of silk and one sticky aggregate for different tasks (Adrianos et al., 2013; Rising and Johansson, 2015). Spider silks have unique properties such as outstanding extensibility, toughness, and tensile strength, which exceed most other natural or synthetic materials such as bone, elastin, silkworm silk, high-tensile steel and synthetic rubber (Askarieh et al., 2010; Hayashi and Lewis, 2001; Guerette et al., 1996; Heim et al., 2010; Meyers et al., 2013). Beyond its excellent mechanical properties, spider silk is also biocompatible and biodegradable, making its use very promising to fulfill various unique demands (Schacht et al., 2015), including some medical applications such as drug delivery vesicles and cell scaffold (Lammel et al., 2011; Wang et al., 2006; Widhe et al., 2013; Wohlrab et al., 2012), and military applications such as body armor and lightweight gear (Lewis, 2006; Teulé et al., 2007). Because of the territorial and cannibalistic nature of the spider it is impossible to extract spider silk on a large scale, therefore researchers attempt to produce artificial spider-silk-like fibers using genetic engineering and study the relationship between spidroin structure and function in order to improve both the amount and quality of the product (Adrianos et al., 2013; Lee et al., 2007).

Flagelliform spidroin (Flag) contains (GPGGX)*n* repeat motifs (43≤*n*≤63, X=A/V/Y/S) that form β-turns, juxtaposed to (GGX)*n* motifs and spacer motifs that form helices and β-sheets. These motifs combine to form spring-like spirals that provide the excellent extensibility of Flag (Adrianos et al., 2013; Hayashi and Lewis, 2001, 2000; Scheibel, 2004). Flag has been widely studied due to its outstanding extensibility based on specific structure and large protein size (over 360 kDa); however, the core domain of Flag is highly repetitive and rich in Gly content (over 50%), resulting in the recombinant Flag being difficult to express in the current engineering systems (Hayashi and Lewis, 2001; Heim et al., 2010; Vendrely and Scheibel, 2007). Therefore, the knowledge of the relationship between protein size and mechanical properties is of urgent demand.

Here, the aim of this study is to illustrate the correlation between the length of the functional repetitive domain and mechanical properties of the synthetic Flag fibers. Four recombinant Flag spidroin with different sizes, like N-Scn-C (*n*=1-4), were constructed, expressed and spun to the silk-like fiber by wet spinning. The spun fiber was subjected to continuous post-drawing by a custom-made device. This study shows that the mechanical properties (such as extensibility and toughness) are improved by the increase of functional repetitive domains. The best elongation is obtained from N-Sc4-C (58.76%), while the highest strength belongs to N-Sc3-C (107.54 MPa). The Raman data illustrates the mechanical test results from the structural respect. This study may indicate the correlation between the size of recombinant spidroin and the mechanical properties of the synthetic Flag fibers, which might be applied for various medical or industrial settings.

## RESULTS

### Flag silk gene

**Fig. 1.**
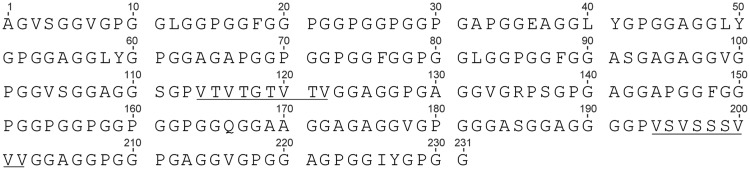
**The amino acid sequence of the flagelliform silk protein Sc (the sequence was shown in one character codon).** Two rich valine blocks are shown on underline.

A Flag repetitive domain gene (Sc) encoding 231 amino acids (Fig. 1) was screened from *Araneus ventricosus* (*A.v.*) genomic library and aligned with the only accessible Flag repetitive domain sequence (*A.v*.Flag-CT) in the database (GenBank: EF025541) (Lee et al., 2007). The sequence identity between Sc and *A.v.*Flag-CT was 91.7% (Fig. S1). The Sc amino acid sequence showed GPGGX and GGX (X=A, V, L, F) motifs were contained, which were also conserved within Flag spidroin from *Nephila clavipes* (*N.c.*) (Hayashi and Lewis, 2000). When compared with *N.c.* Flag repetitive domain, which usually contains 40∼60 pentapeptide (GPGGX) in one repeat domain, our Flag silk gene contained less pentapeptide repetitive motifs (Hayashi and Lewis, 2001; Hayashi and Lewis, 2000). Two valine-rich blocks, VTVTGTVTV and VSVSSSVVV (shown as underlined sequences in Fig. 1), were found in Sc and regarded as the spacer motifs (Lefèvre and Pézolet, 2012). The Flag repetitive gene from *A.v.* contains less conserved pentapeptide repetitive motifs and two small spacer motifs, which was different from the homologous genes of other species such as *Argiope trifasciata* (*A.t.*), *Nephila madagascariensis* (*N.m.*), or *Nephila clavipes* (*N.c.*) (Hayashi and Lewis, 2001).

### Recombinant spidroin expression and purification

**Fig. 2.**
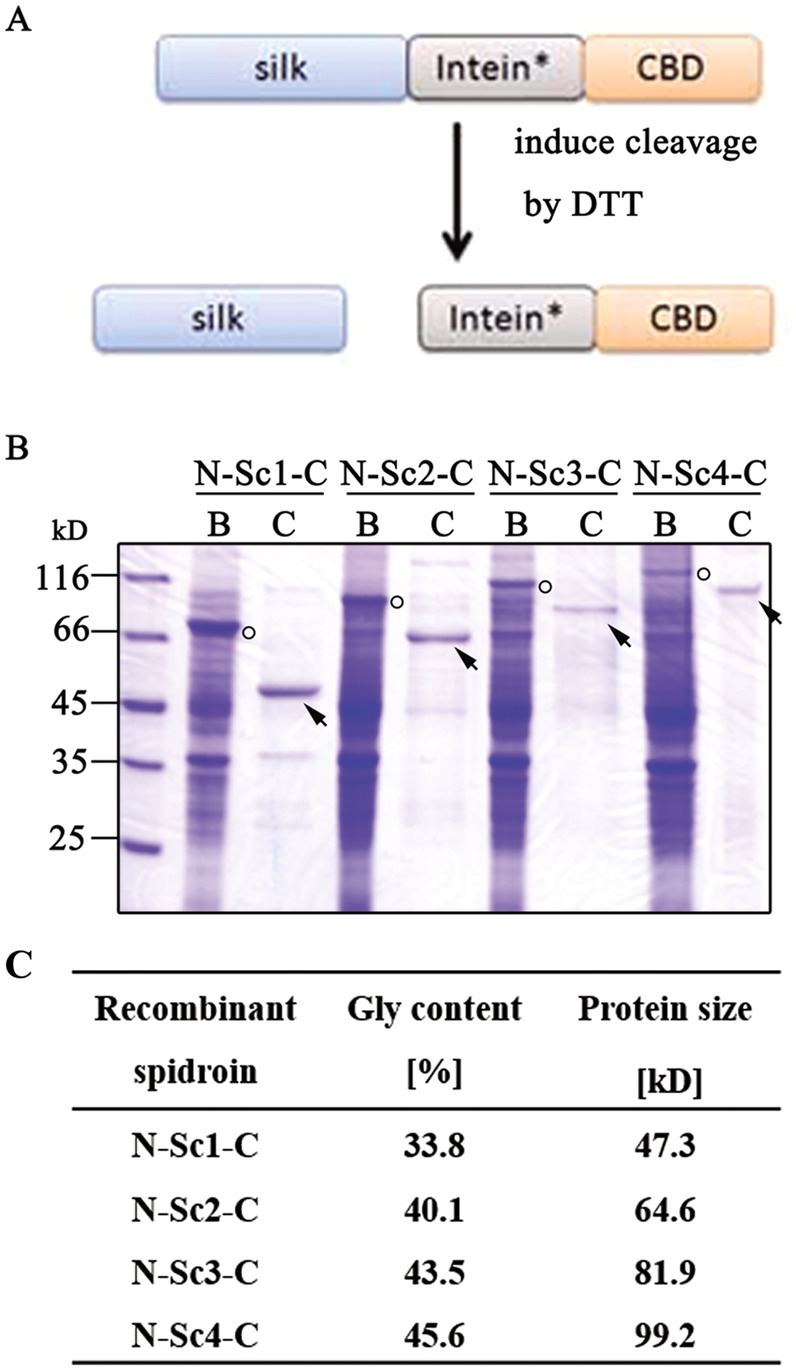
**Purification of the recombinant spidroin.** (A) Mechanism of the purification system. Asterisks indicate the modified intein which can be induced N-cleavage by DTT. (B) SDS-PAGE of the purified protein. The silk precursor proteins and purified proteins were indicated by circles and arrows, respectively. B, before cleavage sample; C, the cleavage products: target protein. (C) The molecular weight and the Gly content of the recombinant spidroin.

To study the relationship between spidroin size and the mechanical properties of recombinant Flag fiber, four recombinant spidroin with different lengths of repetitive domain (Sc) were constructed: N-Sc1-C, N-Sc2-C, N-Sc3-C and N-Sc4-C (shown in Fig. 2C). The conserved non-repetitive domains amino-terminal (NT) and carboxy-terminal (CT) were flanked to the Sc; NT plays functional roles in the spidroin storage and the CT influences the silk formation (Andersson et al., 2014; Kronqvist et al., 2014; Landreh et al., 2010; Wang et al., 2014). Besides, the CT has already been proven to contribute to extensibility in synthetic fibers (Gnesa et al., 2012). Then, the min-spidroin like N-Scn-C (*n*=1-4) was constructed to produce the silk-like fibers. The N/C domains of the recombinant Flag spidroin were chosen from the MaSp2 as their structures and functional roles have already been reported and analyzed (Askarieh et al., 2010; Hagn et al., 2010; Scheibel, 2004).

The intein-mediated purification with chitin binding tag (IMPACT) system was chosen to purify the recombinant spidroin (Fig. 2A) (Evans et al., 1999). N-terminal cleavage system was chosen for production of the recombinant spidroin due to its low pre-cleavage *in vivo* (Mathys et al., 1999). Although the IMPACT system was predominantly performed under native conditions as previously described, it has been reported that the system can also be used under denaturing conditions, and in this study this system was successfully utilized under a denaturing condition. Recombinant spidroin was usually expressed as an inclusion body (IB), especially with high cell density (Liebmann et al., 2008; Zhou et al., 2001). After optimizing the parameters of the IMPACT system (data not shown), the recombinant spidroin can be purified with 4 M urea and 100 mM thio-inducing reagent DTT. The purities of all the proteins were higher than 90% (Fig. 2B). In accordance with previous reports, (Chung et al., 2012; Xia et al., 2010) the yield of the recombinant spidroin decreased with protein size, increasing from ∼30 mg/l of N-Sc1-C to ∼10 mg/l of N-Sc4-C, proving that large size proteins with highly repetitive motifs and Gly content (shown in Fig. 2C) are difficult to express in *E. coli*.

### Preparation of silk

**Fig. 3.**
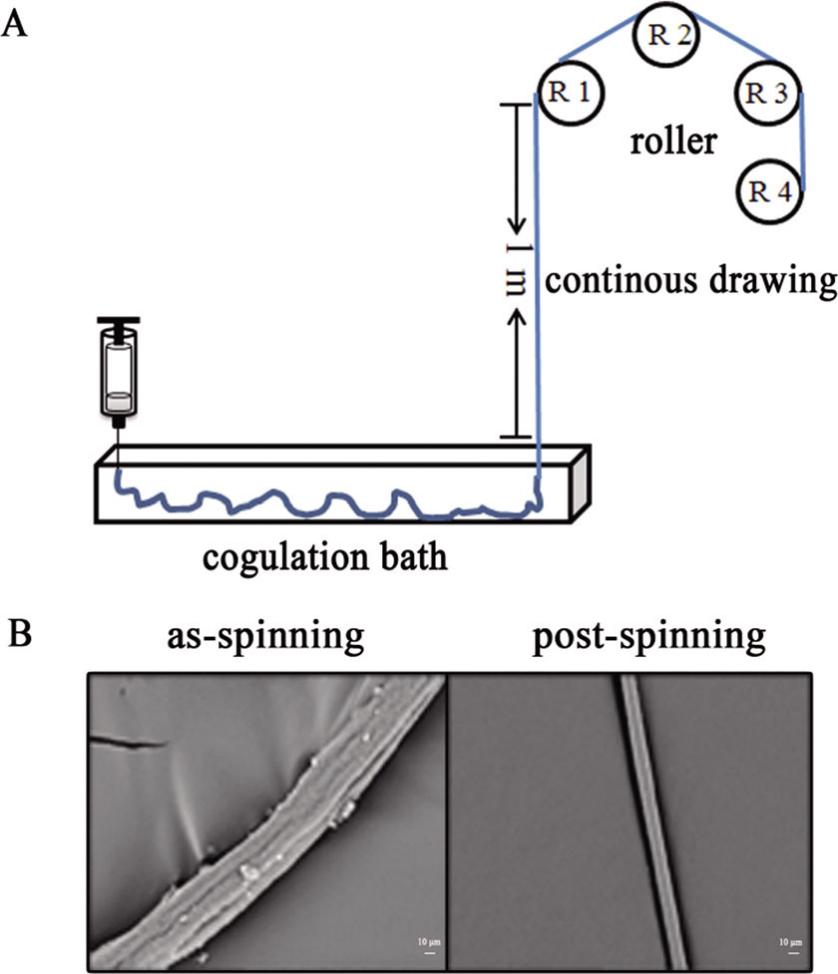
**Post-treatment of the spun fiber.** (A) Continuous post-drawing device. (B) The SEM (scanning electronic microscope) of the spun fiber and post-treatment fiber of N-Sc1-C. Scale bar: 10 μm.

The lyophilized spidroin was re-solubilized in HFIP (Adrianos et al., 2013; Teulé et al., 2007). The spinning dope was injected into a coagulation bath by a pump and the solvent HFIP was subsequently removed to produce silk fibers. Usually, the as-spun fibers need to be treated post-drawing. The importance of the post treating after wet spinning was indicated in many reports (Adrianos et al., 2013; An et al., 2011; Lin et al., 2013; Liu et al., 2005), such as enhancing the orientation co-efficiency of the fibers and decreasing the gap in the fibers during spinning, which affects the mechanical properties of fibers. In this study, we used a custom-made device for the continuous drawing of silk (Fig. 3A). This device was a slightly modified version of Chen's device (Yan et al., 2010). The first three rollers used in this device were not at the same level, which improved the friction between the fiber and the roller. This modification fixed the fibers on rollers and controlled drawing ratio better. The distance between the coagulation bath and roller 1 (R1) was designed as 1 m in order to ensure most of the solvent such as methanol and HFIP be evaporated. The SEM picture showed that the surface of the post-drawing fiber was much smoother than the as-spun fiber with no grooves observed (Fig. 3B). Therefore, our continuous drawing device was proved to be efficient for fibers post-treatment. Further studies could be carried out to optimize the spinning condition and device parameters for large-scale production of silk fibers.

### Morphology of the fibers

**Fig. 4.**
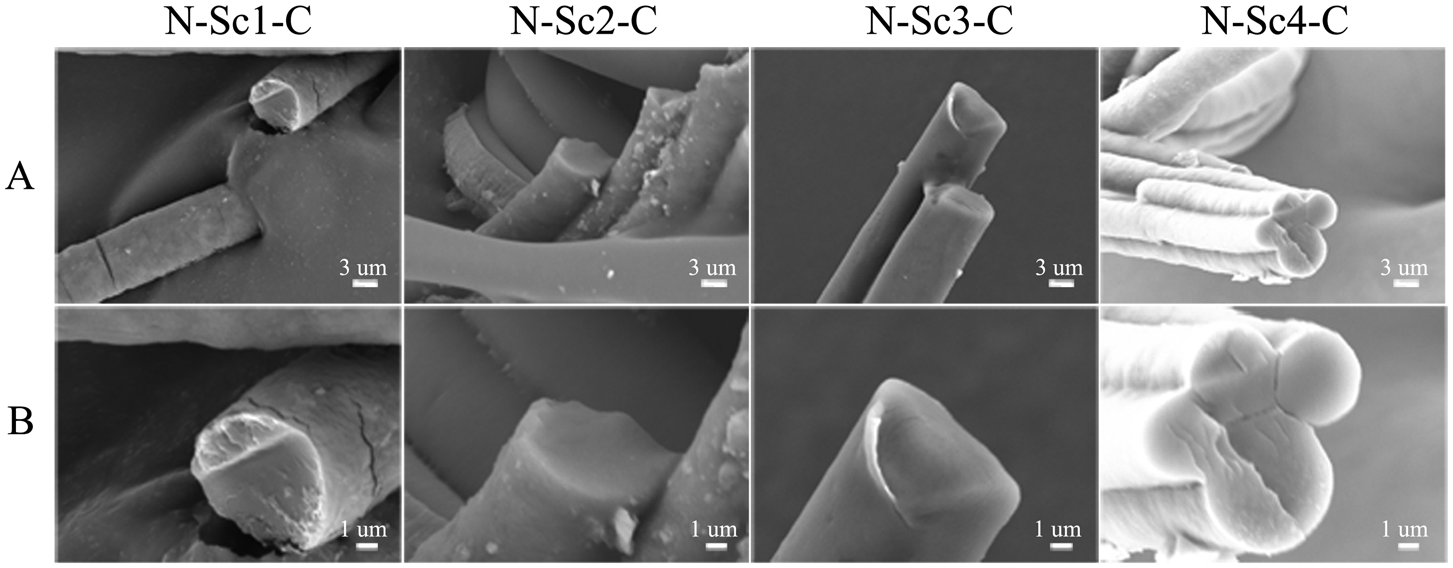
**The inner morphology of the synthetic fibers by SEM under different amplifications.** Scale bars: 3 μm in A; and 2 μm in B.

The inner morphology of the fibers was determined by scanning electronic microscopy (SEM). Fiber samples were immersed in liquid nitrogen and subjected to brittle fracture (Fig. 4). In this process, it was difficult to set a single fiber immersed in liquid nitrogen, take it out, and subject it to brittle fracture; therefore, to obtain the cross section, a large amount of each type of fiber was immersed in liquid nitrogen. As a result, some fibers stuck together (such as N-Sc2-C and N-Sc4-C fibers; Fig. 4A). Besides, the observed cross section located at the fibers' nonuniform position where the diameter was heterogeneous, such as N-Sc3-C fibers and N-Sc4-C fibers (Fig. 4B). This SEM showed that fibers generated by the wet-spinning and continuous drawing method had a smooth cross section without obvious gaps. Previous studies have shown that silk generated from lower-sized proteins had irregular voids (Xia et al., 2010). However, no obvious gaps were observed is this study, even in the smallest protein (N-Sc1-C: 47.3 kDa). The results indicated that emerged irregular voids may be related to the type of spidroin, even the spinning method.

### Mechanical property test

**Fig. 5.**
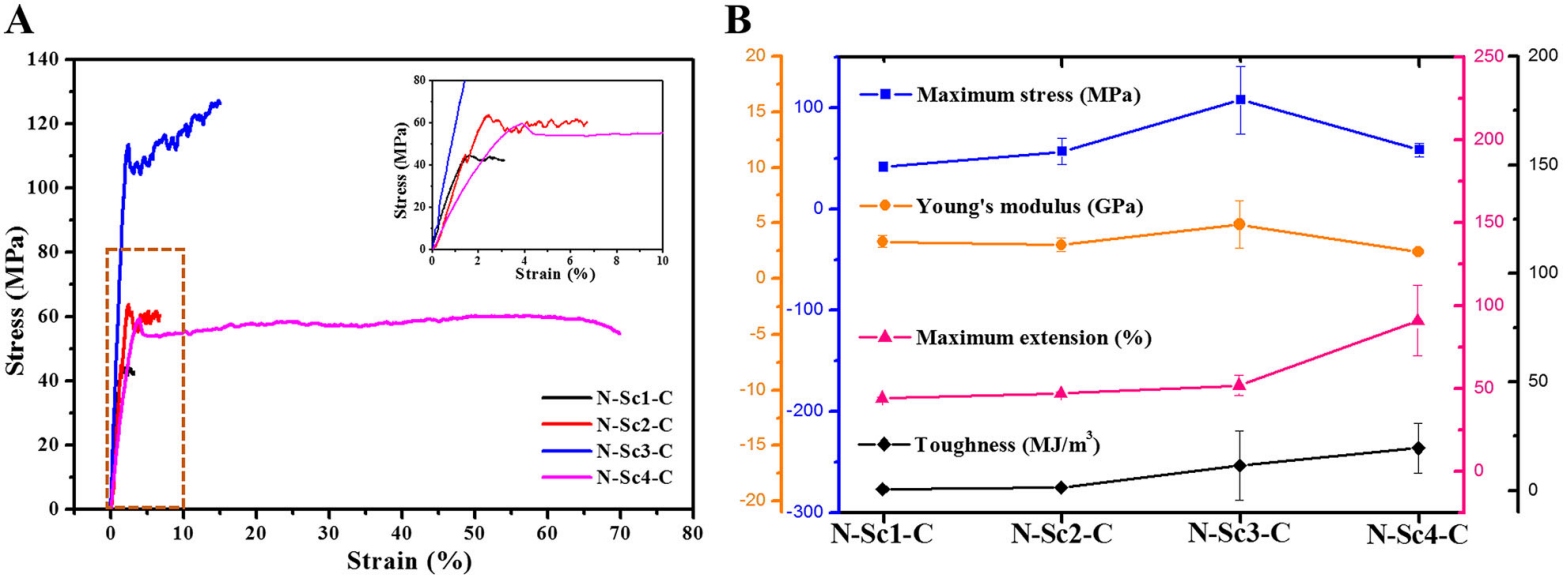
**The mechanical properties of the synthetic fibers.** (A) The stress-strain curves show the traditional features of one of each type of fiber, the embedded chart illustrates the enlargement of the rectangular region (indicated by orange dotted line) of the stress-strain curves. (B) The embedded bar chart illustrates the summary of at least 10 sample testing results. Error bars indicate the standard deviation.

Before the mechanical tests, the extruded fibers were cut and fixed on testing cards, and all samples were checked by the light microscope to ensure the fibers were of uneven thickness. Fig. S2 shows the morphology of one fiber of each type under the light microscope, and the diameter of fibers was calculated. The stress-strain curves and mechanical testing data (maximum stress, maximum extension, Young's modulus and toughness) are shown in Fig. 5 and Table 1, respectively. According to the mechanical testing results, with the increasing of recombinant protein size the breaking strain increased from 2.73% (N-Sc1-C) to 58.76% (N-Sc4-C). The increase of size (the size of Sc: 17.3 kDa) between two adjacent recombinant spidroins was the same from N-Sc1-C to N-Sc4-C (shown in Fig. 2C). The breaking strain increased slowly from N-Sc1-C (2.73%) to N-Sc3-C (11.77%), until a big leap from 11.77% to 58.76% occurred between N-Sc3-C (81.9 kDa) and N-Sc4-C (99.2 kDa). However, the breaking stress of N-Sc4-C (58.39 MPa) unexpectedly decreased compared to N-Sc3-C which possessed the highest strength of 107.54 MPa. A recent report indicated that the larger sized spidroin (from MaSp) possessed better mechanical properties including maximum stress, maximum extension, Young's modulus (Xia et al., 2010). A discrepancy occurs here when comparing to this previously reported data, which may be due to the differences among the repetitive domain of various spidroins (Table S1) (Hayashi and Lewis, 2001). The spinning method may also contribute to this discrepancy, and requires further study (Haq and Khan, 2005; Teulé et al., 2007). Interestingly, N-Sc3-C had the smallest diameter (4.31 μm) but the best maximum stress (107.54 MPa) and Young's modulus (4.89 GPa) (shown in Table 1) compared to the other types of fibers. The toughness of synthetic fibers increased with ascending repeat length (N-Sc1-C and N-Sc4-C was increased from 0.49 to 19.46 MJ/m^3^; Fig. 5B). We speculated that the overall structure of the synthetic fibers which spun from the larger spidroin may be more organized, though the HFIP may influence the secondary structure formation. The toughness of synthetic fibers was lower than the native flagelliform silk, which is about 150 MJ/m^3^ (Gosline et al., 1999; Teulé et al., 2007). This can be explained by the fact that the recombinant spidroin are much smaller than the native silk proteins (larger than 360 kDa) and the spinning method affect the properties of the fibers.

**Table 1.**
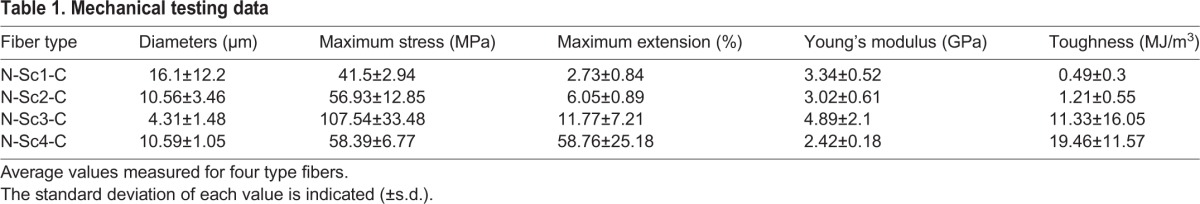
**Mechanical testing data**

### Fiber structure analysis

**Fig. 6.**
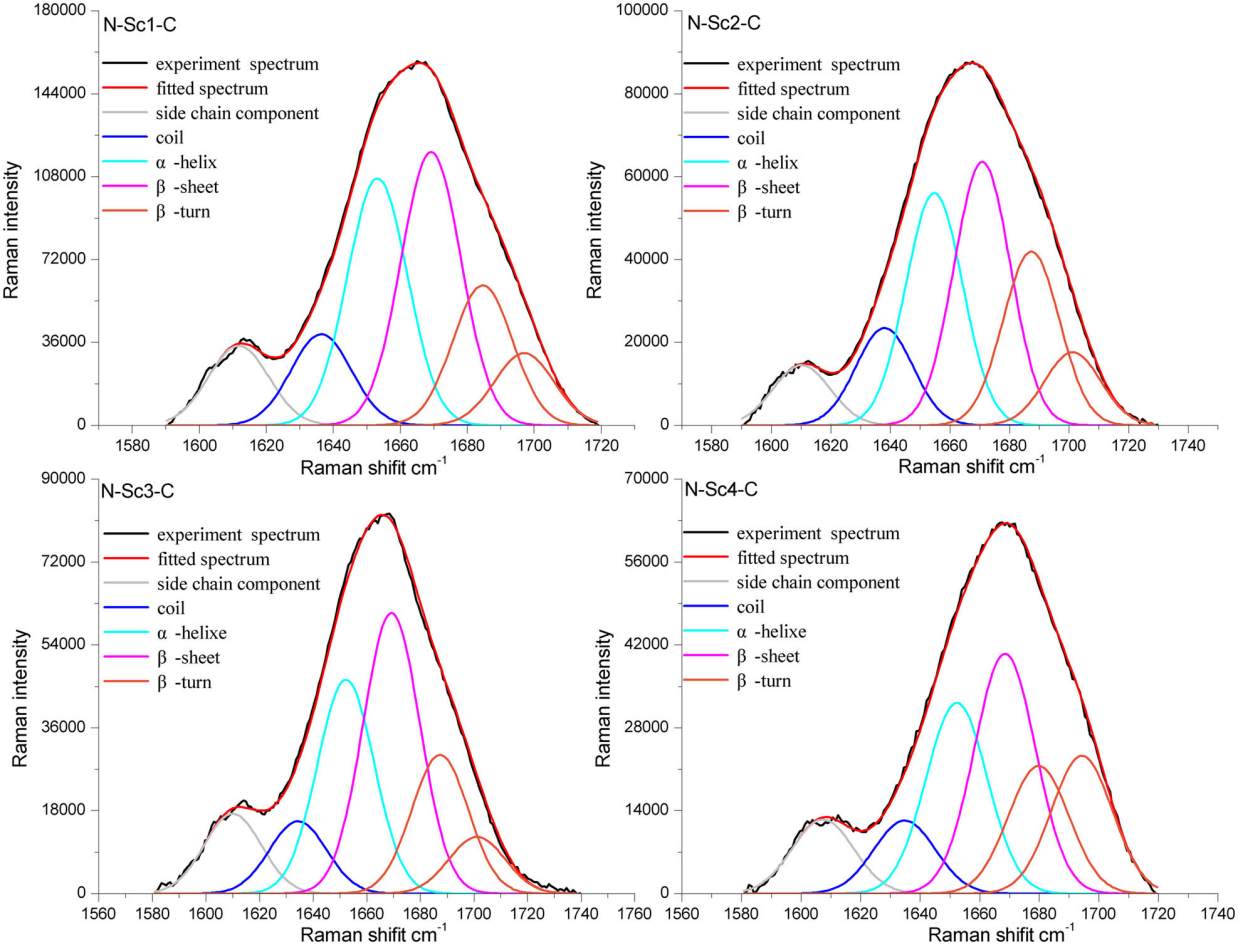
**Raman spectra of the synthetic fibers.** The amid І was divided into five peaks via Peak fit software. The charts show the Raman spectra of one of each type of fiber in amid І region. Experimental spectrum, fitting spectrum and the divided spectrum were showed in different colors.

The micro-Raman spectroscopy system was adopted to determine the secondary structure of micron-sized silk. The amide I data was analyzed as previously reported using the peak-fit software, dividing it into five peaks (Lefèvre et al., 2011, 2007; Tucker et al., 2014; Xu et al., 2012). The secondary structure has the characteristic peak in the amide I; the disordered structure has the peak near 1639 cm^−1^; the α-helix has the peak near 1656 cm^−1^; the β-sheet has the peak near 1669 cm^−1^; the β-turn has the peak near 1684 cm^−1^ and 1698 cm^−1^. Fig. 6 shows the spectral decomposition in the amide I region of one of each type of synthetic fiber. The r^2^ values of the peak fitting for four types of fibers were nearly 1, proving the reliability of the structure predictions (N-Sc1-C: r^2^=0.99926; N-Sc2-C: r^2^=0.99857; N-Sc3-C: r^2^=0.99061; N-Sc4-C: r^2^=0.99919). From the divided peaks, the secondary structural fractions in fibers could be calculated via accounting the area of the peak (shown in Table 2). The content of the unfolded structure did not show significant differences among four fibers, but was relatively higher than the natural spider silks (Holland et al., 2008; Lefèvre et al., 2007). The method of fiber production may contribute to the high crystallization level. The compositions of the different structures in the four fiber types were consistent with the results from mechanical tests (shown in Table 2). The content of β-turn in N-Sc4-C (31.47%) was the highest, which contributed to its highest extensibility; while β-sheet was the highest in N-Sc3-C (38.34%) which contributed to the stiffness of the silk, resulting in N-Sc3-C possessing the highest breaking stress (107.54 MPa; shown in Fig. 5). These secondary structures revealed by Raman data analysis may be related to the differences of mechanical properties among four fiber types and provide deeper insights into the future property prediction of a defined fiber.

**Table 2.**
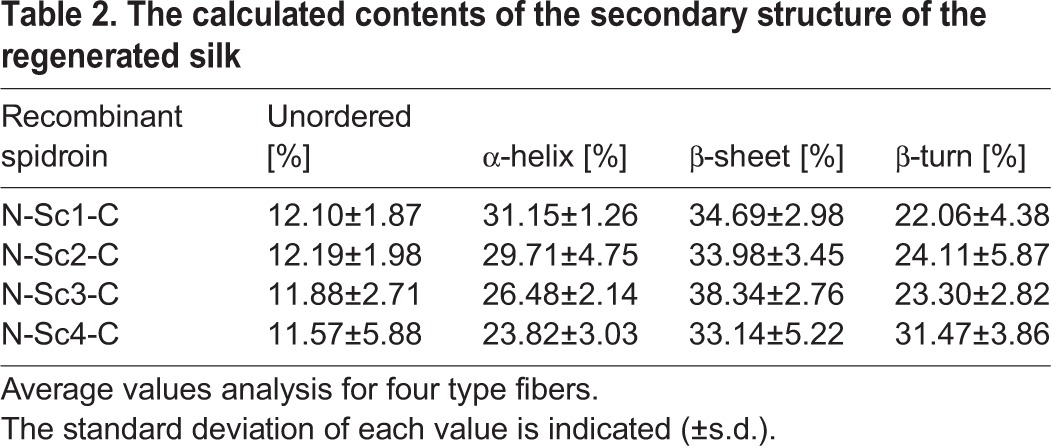
**The calculated contents of the secondary structure of the regenerated silk**

## DISCUSSION

In this study, four recombinant spidroins (N-Scn-C, *n*=1, 2, 3, 4) were constructed with the N/C non-repetitive domains (∼15 kDa, from MaSp2) and the repetitive domain Sc (∼17.3 kDa, from flagelliform). Meanwhile, another construct Sc1 was constructed without the terminal domain N/C as a control. The CD spectra of the Sc1 and N-Sc1-C were done to detect their secondary structure in PB buffer and HFIP (shown in Fig. S3). From the CD spectra we speculated that the N/C domain plays a functional role for the structure of the Sc1 in PB buffer or HFIP, and the HFIP may affect the protein structure. Though HFIP may change the protein structure, some reports proved that the conserved C-terminal domain of spider tubuliform spidroin 1 contributed to extensibility in synthetic fibers even in HFIP (Gnesa et al., 2012). In future studies, we will do more work to investigate the role of the N/C terminal domain in the synthetic fibers in HFIP.

In the small recombinant spidroin N-Sc1-C, the N/C domain dominated the protein structure and the properties of fiber, leading to a lower extensibility (2.73%) and breaking stress (41.5 MPa). With the increase of repetitive domain Sc, which contains many GPGGX and GGX motifs contributing to extensibility (Adrianos et al., 2013), the properties of Sc became dominant within the total fiber properties. Thus, based on the mechanical testing results, we concluded that the extensibility of the fibers increased with the increasing length of repetitive domain. Nevertheless, there was a leap between the extensibility of N-Sc3-C (11.77%) and N-Sc4-C (58.76%) which accounted for nearly one third of the native flagelliform silk (200%). This may be caused by the structure and size difference. According to the structure analysis the N-Sc4-C had a high level of β-turn structure (31.47%) compared to others, and a large molecule weight (99.2 kDa) which may form a regular structure similar to the native Flag silk, which were usually larger than 360 kDa.

Interestingly, the N-Sc3-C fibers had the smaller diameter, though they showed the best strength. We speculated that the moderate molecule weight of N-Sc3-C spidroin supplies better overall organization. This may cause its diameter to be smaller than the others and contain the most β-sheet content (38.34%), providing a higher strength. Besides, some unknown mechanisms might exist resulting in these unusual changes. Further studies on these mechanisms may help to produce fibers that meet the specific requirements in various settings. Some reports also claim that synthetic fibers with small diameters have better properties than others with a large diameter (Teulé et al., 2007). Apart from the highest extensibility of the N-Sc4-C, N-Sc3-C had a higher breaking stress of 107.54 MPa and reasonably high extensibility (11.77%) compared with other recombinant spidroin which had the similar molecular weight (Xia et al., 2010). Therefore, the N-Sc3-C fibers we constructed in this study may have excellent mechanical properties regarding both extensibility and strength which might be designed for specific applications.

The mechanical tests and Raman data analysis indicated there might be a molecular weight threshold of the functional repetitive domain relating to extensibility of the silk. In this study, the largest protein (N-Sc4-C: 99 kDa) possessed the highest extensibility. When compared to the native Flag silk with >200% extensibility, our constructed fibers were lower since the native Flag silk proteins were usually larger than 360 kDa (Hayashi and Lewis, 2000). This further proves the positive correlation we proposed here between protein size and extensibility. In this study we only constructed and analyzed four recombinant spidroins with different molecule weight due to the low yield of large protein under laboratory conditions, with a highly repetitive sequence and rich Gly content (Teulé et al., 2007). From the extensibility data analysis both from artificial fibers and natural silks, we speculated that the molecular weight threshold of the repetitive domain for good extensible synthetic fibers may be at least 52 kDa (3 Sc repetitive domain here), which might be used as basic parameter for designing artificial fibers with outstanding extensibility.

### Conclusion

In this study, we investigated the correlation between the recombinant spidroin size and the synthetic fiber properties. Besides, the relationship between the structure and function of repetitive domain in synthetic fibers was also studied. Four different sizes of recombinant spidroin were constructed and generated into fibers by wet-spinning using a custom-made post-drawing device. The fiber morphology, mechanical properties, and secondary structure were comprehensively evaluated. The results indicated that the extensibility of the synthetic fibers was in positive correlation with the recombinant Flag spidroin size, and may imply a potential threshold of the protein size for excellent extensibility of the fibers (in this study, N-Sc4-C). Furthermore, our study showed the relationship between the function and structure of the synthetic fibers, which was useful for functional protein design with various extensibility. In addition, these synthetic fibers from Flag spidroin with acceptable mechanical properties may provide a promising potential as the biomaterial for specific applications.

## MATERIALS AND METHODS

### Materials

Chemical reagents and kits were purchased from Sangon (Shanghai, China). All restriction enzymes were purchased from New England BioLabs (Ipswich, MA, USA). The gene of the Flag repetitive domain was screened from an *Araneus ventricosus* (*A.v.*) genomic library (Chen et al., 2012), and the amino acid sequence was shown in Fig. 1. The gene codon was adjusted to *E. coli* (designated as Sc1, completed by Genway company, Shanghai, China) and two enzyme recognition sites, *Bsa*І and *Bfu*AІ, were added to amino-terminal (N) and carboxy-terminal (C) of the silk gene in pET32, respectively, to make clone pETSc1 with *Nde*І and *Xho*І. The N and C terminal non-repetitive domains were obtained according to the major ampullate spidroin 2 (MaSp2) of *Argiope trifasciata* and *Argiope aurantia*, by using *E. coli* gene codon (Genebank: AAZ15371.1 and AAK30592.1, respectively).

### Cloning strategy

The cloning strategy was performed as previously described (Huemmerich et al., 2004). Briefly, pETSc2 was constructed by digesting pETSc1 with *Bsa*I/*Pst*I and *Bfu*AI/*Pst*I followed by Sc1 insertion. The same procedures were used to generate pETSc3 and pETSc4. The N terminal non-repetitive domain was amplified by primers N-1 (5′-GGGAATTCATATGGGTGAACAAGGTGGTCTGTC-3′) and N-2 (5′-ACCGGTGGTCTCGCCAGCGTACGGGCCACGACCGCCTG-3′); while C terminal non-repetitive domain was amplified by primers C-1 (5′-GGCACCTGCTTCTGGCGGTGGTCAACGTGGTCCTCGCTC-3′) and C-2 (5′-GGCCTCGAGGCTCTTCCGCAACCCAGAGCCTGAGTCAG-3′). Underlining shows the site of restriction enzymes, *Nde*I, *Bsa*I, *Bfu*AI, *Xho*I, respectively. The N and C non-repetitive domains were cloned into the pETSc by *Nde*І/*Bsa*І and *Bfu*AІ/*Xho*І, resulting in pET-N-Sc1-C, pET-N-Sc2-C, pET-N-Sc3-C and pET-N-Sc4-C, respectively.

To construct the expression vector, the recombinant plasmids pET-N-Scn-C (*n*=1-4) were digested by *Nde*І and *Sap*І and ligated into pTWIN1 resulting in pT-N-Sc1-C, pT-N-Sc2-C, pT-N-Sc3-C and pT-N-Sc4-C, respectively. All the recombinant plasmids were sequenced.

### Protein expression and purification

All expression clones (pT-N-Scn-C) in BL21 (DE3) cells were cultured overnight in 10 ml of Luria broth (LB) containing 100 μg/ml ampicillin in a shaking incubator at 37°C. The overnight culture was inoculated into 1 liter of LB containing 100 μg/ml ampicillin. When the OD_600_ reached 0.6∼0.8, the cultures were induced by addition of isopropyl β-D-1-thiogalactopyranoside (IPTG) to a final concentration of 0.8 mM. Upon induction, the temperature was decreased to 25°C for 16 h. The collected overnight cells were re-suspended in a lysis buffer (20 mM Tris.Cl, 500 mM NaCl, 8 M urea, pH 8.0) by 20% (w/v), lysed by a pressure homogenizer JN-3000 plus (JNBIO, Guangzhou, China) at 1300 bar, and was further incubated for an additional 1 h at room temperature. Cell lysates were clarified by centrifugation at 21,000×***g*** for 20 min and the supernatants were dialyzed in a column buffer (4 M urea, 500 mM NaCl, 20 mM Tris.Cl, pH 8.0).

The purification of the silk proteins was performed according to the N-terminal cleavage protocol of the intein mediated purification with an affinity chitin-binding tag (IMPACT) system (New England Biolabs). All the purification buffers contained 4 M urea and the elution buffer added 100 mM DTT additionally. The IMPACT system carries an intein-chitin binding domain (intein-CBD) tag for one-step purification using a chitin resin. The target protein can be specifically released from the tag through induction of intein cleavage by the addition of a thiol-containing buffer (we used DTT) and elute from the column. Thus, the target protein can be collected without any extra tag. The purified recombinant spidroin was dialyzed in pure water to remove salts and centrifugation and freeze-drying.

### Fiber spinning

The lyophilized proteins were re-solubilized in HFIP (hexafluoroisopropanol) to make a 15∼20% (wt/v) spinning drop. The spinning drop was moved into a syringe and injected by a pump (KD Scientific, Ringoes, NJ, USA) into a coagulation bath. The spinning rate was 20 μl/min and the coagulation solvent was 90% methanol (v/v). The silk-like fiber was spun and drawn from the bath by a custom made device (Fig. 3). There was a distance from the spinning nozzle to the position where the fibers were drawn out from the coagulation bath (the length of the vessel of coagulation bath is about 60 cm). During this whole process, the fibers were guided with a tweezers until the fibers began twining on the rollers. The rates of roller 1, 2, 3, and collecting roller 4 were 2.4 m/min, 3.6 m/min, 4.8 m/min, and 6 m/min, respectively. The collected fibers were dried in a sealed box with desiccant for at least 48 h to prevent contraction and maintain the extended length for the measurements.

### Properties testing

Before the mechanical tests, all fibers were fixed on testing cards and checked by the light microscope to ensure the fibers were of uneven thickness. The diameters of fibers were measured by Image Tool software. The mechanical tests of the synthetic fibers were conducted on a UTM T150 (universal testing machine) from Agilent. The gage length was 6 mm and the starting load strength was 750 μN. A minimum ten samples were tested for each type of silk.

The secondary structures of the fibers were determined using Micro-Raman Spectroscopy System (inVia-Reflex, Renishaw, Gloucestershire, UK). For each sample, at least seven fibers were tested to assess sample uniformity, and the spectral decomposition of the amide I region was analyzed by the peak-fit software to calculate the secondary structure composition (Cranes Software International Ltd., San Jose, CA, USA).
